# Supplementary material for: An open-source framework for large-scale, flexible evaluation of biomedical text mining systems
Source: J Biomed Discov Collab. 2008 Jan 29;3:1. doi: 10.1186/1747-5333-3-1 (PMC2276192; doi:10.1186/1747-5333-3-1)
Supplement: Additional file 1 — Evaluation platform source code. The additional file contains the software evaluation framework discussed in this paper. The most current release can be downloaded from SourceForge.net [1]. See the accompanying README file for instructions on installation and use. Also included in the distribution is a collection of UIMA wrappers for some commonly used BioNLP tools and annotated corpora. [file 1747-5333-3-1-S1.zip › bionlp_uima_repository-1.0.1/docs/api/edu/uchsc/ccp/util/external/lingpipe/LingPipe_Util.html]

LingPipe\_Util (Center for Computational Pharmacology Util/UIMA API)


|  |  |  |
| --- | --- | --- |
| **Overview** \| **Package** \| **Class** \| **Tree** \| **Deprecated** \| **Index** \| **Help** | |  |
| PREV CLASS   NEXT CLASS | **FRAMES**    **NO FRAMES**     **All Classes** |  |
| SUMMARY: NESTED \| FIELD \| CONSTR \| METHOD | DETAIL: FIELD \| CONSTR \| METHOD |  |


---


## edu.uchsc.ccp.util.external.lingpipe Class LingPipe\_Util

```
java.lang.Object
  edu.uchsc.ccp.util.external.lingpipe.LingPipe_Util
```

---

``` public class LingPipe_Util extends Object ```

---

**Constructor Summary**

`LingPipe_Util()`


| **Method Summary** | |
| --- | --- |
| `static void` | `evaluateSentenceDetector(String evaluationCorpusLocation, String errorFilePath)`             Evaluate the LingPipe Sentence detector Taken from: http://alias-i.com/lingpipe/demos/tutorial/sentences/src/SentenceModelEvaluator.java |
| `static List<TextAnnotation>` | `extractEntities(String inputText, String documentID)`             Use with Lingpipe\_3.0.0 |
| `static List<TextAnnotation>` | `findSentences(String inputText, String documentID)`             Use SentenceModel to find sentence boundaries in text LingPipe relevant code used by this method was taken from: http://alias-i.com/lingpipe/demos/tutorial/sentences/src/SentenceChunkerDemo.java |
| `static int` | `getAnnotatorID()` |
| `static Map<String,List<String>>` | `getGeniaEntitiesFromParse()`             Returns a mapping between sentence text and entity sets from the previously parsed Genia input text. |
| `static List<String>` | `getGeniaSentencesFromParse()`             Returns a list of sentences that were extracted from the previously parsed Genia input text |
| `static void` | `initializeEntityTaggingSystem(String modelFile, String entityType)`             Use with Lingpipe\_3.0.0 |
| `static void` | `initializePOSTaggingSystem(String modelFile)`             Ready the system for POS tagging. |
| `static void` | `parseGeniaText(String geniaText)`             parse input text that is in the GENIA (XML-embedded entity) format |
| `static ArrayList<TextAnnotation>` | `runPOSTagger(String inputText, String documentID)`             Much of this code was taken from: http://alias-i.com/lingpipe/demos/tutorial/posTags/src/RunMedPost.java NOTE: initializeTaggingSystem() must be called before this method. |
| `static void` | `trainGENIATagger(String GENIACorpusLocation, String outputModelFileLocation)` |
| `static void` | `trainMedPostTagger(String medPostCorpusLocation, String outputModelFileLocation)`             Much of this code was taken from: http://alias-i.com/lingpipe/demos/tutorial/posTags/src/TrainMedPost.java |
| `static void` | `warn(String message)` |

**Methods inherited from class java.lang.Object**

`clone, equals, finalize, getClass, hashCode, notify, notifyAll, toString, wait, wait, wait`

**Constructor Detail**

### LingPipe\_Util

```
public LingPipe_Util()
```


**Method Detail**

### findSentences

```
public static List<TextAnnotation> findSentences(String inputText,
                                                 String documentID)
```

:   Use SentenceModel to find sentence boundaries in text LingPipe relevant code used by this method was taken from:
    http://alias-i.com/lingpipe/demos/tutorial/sentences/src/SentenceChunkerDemo.java

    :   **Parameters:**: `inputText` -

---


### trainMedPostTagger

```
public static void trainMedPostTagger(String medPostCorpusLocation,
                                      String outputModelFileLocation)
                               throws IOException
```

:   Much of this code was taken from: http://alias-i.com/lingpipe/demos/tutorial/posTags/src/TrainMedPost.java

    :   **Throws:**: `IOException`

---


### trainGENIATagger

```
public static void trainGENIATagger(String GENIACorpusLocation,
                                    String outputModelFileLocation)
                             throws IOException
```

:   **Parameters:**: `GENIACorpusLocation` -: `outputModelFileLocation` - **Throws:**: `IOException`

---


### runPOSTagger

```
public static ArrayList<TextAnnotation> runPOSTagger(String inputText,
                                                     String documentID)
```

:   Much of this code was taken from: http://alias-i.com/lingpipe/demos/tutorial/posTags/src/RunMedPost.java NOTE:
    initializeTaggingSystem() must be called before this method.

    :   **Parameters:**: `medPostModelFile` -: `inputText` -

---


### initializePOSTaggingSystem

```
public static void initializePOSTaggingSystem(String modelFile)
                                       throws ClassNotFoundException,
                                              IOException
```

:   Ready the system for POS tagging.

    :   **Throws:**: `ClassNotFoundException`: `IOException`

---


### getAnnotatorID

```
public static int getAnnotatorID()
```

---


### evaluateSentenceDetector

```
public static void evaluateSentenceDetector(String evaluationCorpusLocation,
                                            String errorFilePath)
                                     throws SAXException,
                                            IOException
```

:   Evaluate the LingPipe Sentence detector Taken from:
    http://alias-i.com/lingpipe/demos/tutorial/sentences/src/SentenceModelEvaluator.java

    :   **Throws:**: `SAXException`: `IOException`

---


### initializeEntityTaggingSystem

```
public static void initializeEntityTaggingSystem(String modelFile,
                                                 String entityType)
                                          throws IOException
```

:   Use with Lingpipe\_3.0.0

    :   **Throws:**: `IOException`

---


### extractEntities

```
public static List<TextAnnotation> extractEntities(String inputText,
                                                   String documentID)
```

:   Use with Lingpipe\_3.0.0

---


### parseGeniaText

```
public static void parseGeniaText(String geniaText)
                           throws SAXException,
                                  IOException
```

:   parse input text that is in the GENIA (XML-embedded entity) format

    :   **Parameters:**: `geniaText` - **Throws:**: `SAXException`: `IOException`

---


### getGeniaSentencesFromParse

```
public static List<String> getGeniaSentencesFromParse()
```

:   Returns a list of sentences that were extracted from the previously parsed Genia input text

    :   **Returns:**

---


### getGeniaEntitiesFromParse

```
public static Map<String,List<String>> getGeniaEntitiesFromParse()
```

:   Returns a mapping between sentence text and entity sets from the previously parsed Genia input text. The entities take the form of
    "spanStart-spanEnd: classMentionName"

    :   **Returns:**

---


### warn

```
public static void warn(String message)
```


---


|  |  |  |
| --- | --- | --- |
| **Overview** \| **Package** \| **Class** \| **Tree** \| **Deprecated** \| **Index** \| **Help** | |  |
| PREV CLASS   NEXT CLASS | **FRAMES**    **NO FRAMES**     **All Classes** |  |
| SUMMARY: NESTED \| FIELD \| CONSTR \| METHOD | DETAIL: FIELD \| CONSTR \| METHOD |  |


---
